# Supplementary material for: Millimeter-wave to near-terahertz sensors based on reversible insulator-to-metal transition in VO2
Source: Commun Mater. 2023 May 22;4(1):34. doi: 10.1038/s43246-023-00350-x (PMC11041681; doi:10.1038/s43246-023-00350-x)
Supplement: Supplementary file 1 — Supplementary Information [file 43246_2023_350_MOESM1_ESM.pdf]

Supplementary information for

Millimeter-wave to near-terahertz sensors based on reversible insulator-to-metal transition in VO_2

Fatemeh Qaderi[1,*], Teodor Rosca[1], Maurizio Burla[2], Juerg Leuthold[2], Denis Flandre[3], and Adrian M. Ionescu[1,*]

[1]Nanoelectronic devices laboratory (Nanolab), Department of Electrical Engineering, École polytechnique fédérale de Lausanne (EPFL), EPFL STI IEL NANOLAB, ELB 335, Station 11, Lausanne, 1015, Switzerland
[2]Institute of Electromagnetic Fields (IEF), Eidgenössische Technische Hochschule Zürich (ETHZ), ETZ K 82, Gloriastrasse 35, Zürich, 8092, Switzerland
[3]ICTEAM, Ecole Polytechnique de Louvain (UCLouvain), ELEN, Place du Levant 3/L5.03.02, Louvain-la-Neuve, 1348, Belgium
[*]fatemeh.qaderi@epfl.ch
[*]adrian.ionescu@epfl.ch

Supplementary Note 1: Operational range of the spike generator

We sweep the gate voltage in order to realize the operational region of the spike generator. It is worth noting that the circuit shows a hysteretic function of the gate voltage, in terms of the initial and final points of the gate voltage which supports oscillating conditions. Supplementary Fig. 1 shows this sweep to find the operating points.

Supplementary Note 2: Setup Considerations

While applying the RF signal to the antennas, the output power of the vector network analyzer (VNA) port connected to the Vanadium dioxide (VO_2) patch was set to the lowest level at -50 dBm, and only the power level from the other port was looked upon. Therefore, the receiving port could be assumed as passive, with no intervening or direct injecting of power to the sensitive VO_2 part. Same measures were applied when connecting the ports to the coplanar waveguide (CPW). More information about the VNA setup can be found here:

220 GHz setup datasheet

Note. Every set of measurements applying RF signal are made with no displacement of the probes, whereas for higher temperatures the displacement was inevitable, which leads to very slight differences in S parameters under same other circumstances.

The dynamics of the sensor depend on the readout method. We have proposed two methods here, direct measurement of the threshold voltage based on VO_2 DC I-V characteristics, or the frequency measurement of the oscillator (AC method). Response time based on DC method: each measurement in this method is to locate the insulator-metal transition (IMT) threshold voltage. Hence, it depends on the speed of sweeping the DC voltage across the expected range of IMT voltages. For the proposed device of this paper, we have the range between 1.5 V to 1.7 V. The voltage resolution will determine the integration time. For a synthesized triangular signal, our oscilloscope operates at 2 GigaSamples/sec (InfiniiVision 1000 X-Series Oscilloscopes).

We can set the slope for the voltage to progress at 1 mV/ns so that we have a resolution of <1 mv at this sampling rate. Therefore, a sweep of 200 mV takes 200 ns. The setup will be the combination of a normal resistor in series with the VO2 impedance and the sampling would be from the shared node between the two. Each acquisition (of a measurement cycle) will take 200 ns, while this data needs to be analyzed offline, to extract the IMT threshold voltage for each cycle. One also needs to wait for the reset time in case of multiple acquisitions, which is at least a microsecond[1].

Response time based on AC method: in the VO2-based astable circuit we can scale the frequency of oscillations as high as the circuit operating point stays within a margin to maintain astability. This margin is achievable through the measurements discussed in the supplementary note 4: "operational range of the spike generator". We used typical values for the circuit components: 150 nF capacitor along with the VO_2 impedances of 1.4 kΩ and 60 Ω for "off" and "on" states, respectively. That leads to a free-running oscillation frequency around 3250 Hz.

The period of each cycle provides a sampled data point. Being exposed to the radiation, the periods go shorter, so the maximum waiting time to collect a single data point would be $\frac{1}{3250Hz} = 308\,\mu s$.

For the bandwidth, we consider the VO_2 switching response time, which is order of a nanosecond for IMT and order of a microsecond for MIT[1]. Along with the circuit design parameters, one could tune the free-running oscillation frequency. According to the ratio of threshold voltage displacement and therefore the frequency shift influenced by the incoming wave power, one can estimate the bandwidth needed for the variation of f_{osc}. In this case we have the reference frequency at 3250 Hz with a frequency shift of 350 Hz corresponding to the maximum power we applied (0 dBm). On top of all, the integration time in this case determines the duration of each acquisition. If we aim for the minimum NEP, we have to use more cycles of the same measurement, at least about 100 ms according to Allan method for this experiment: fig. 6 (c) of the paper.

The output RF frequency of the VNA is up-converted by mm-wave modules (multipliers) connected as extensions to it. The high-frequency signal is then directed to the CPW through GSG infinity probes.

The whole setup for the astable is revealed in Supplementary Fig 2(a). This setup is associated with fig. 1 (c) of the paper. The wafer including VO_2 samples along with the infinity probes are shown in Supplementary Fig. 2(b)

The temperature reservoir along with the setup is also shown in Supplementary Fig. 3. The temperature of the metallic stage (chuck) is controlled this way.

Supplementary Note 3: Antennas Considerations

The same coupled antennas are simulated using finite element method (FEM). The variation of S parameters with respect to the conductivity of VO_2 patch in the middle of one antenna follows the pattern shown in Supplementary Figs. 4(b) and 4(a)

In the antennas configuration of fig. 1 of the paper, only the receiver antenna is VO_2-loaded and DC-biased. The emitter antenna is only identical in the metallic pattern and no bias is applied to it. Port 1 is connected to the emitter, and its own return loss is revealed in fig. 2 (c) of the paper. The notch at 145 GHz shows the impedance match of this individual antenna to the ambient space around it. We should also notice that the return loss of the receiver antenna at port 2 is not the same as the one of port 1 due to the effect of VO_2 leakage on the antenna's electromagnetic configuration. This is shown in Supplementary Supplementary Fig. 5(a) where the peaks from $|S_{21}|$ and $|S_{11}|$ do not happen at the same frequencies.

$|S_{21}|$ represents the matching between the two antennas for a small portion of the power delivered by the emitter antenna to the outer space, i.e. about -10 dB at its maximum according to fig. 2 (d) of the paper. The rest of the power is either absorbed by the ambient, or reflected back to the emitter antenna. In an optimized design, the resonance frequency for the return loss would be tuned exactly on the one of the insertion loss. In fig. 2 (d) we have at least three ranges where $|S_{21}|$ reaches its highest values: 90 GHz, the range 120-150 GHz, and the range 200-220 GHz. These mutual partial-resonances between the two antennas are not necessarily at the same frequency of the individual antenna resonances, as a big portion of the power is already absorbed by the ambiance. The amount of absorption is calculated by $1 - |S_{11}|^2 - |S_{11}|^2$ and illustrated in Supplementary Fig. 5(b). The absorption is considerably high compared to the portion received by the other antenna, and this explains why the $|S_{21}|$ and $|S_{11}|$ curves are not perfectly complementary. On the other hand, the $|S_{21}|$ is more complementary to the $|S_{22}|$.

In the biased antennas, we measured S parameters at different bias voltages. When applying the smallest power (-30 dBm), even the combination with a bias voltage up to 1.705 V does not lead to a phase transition (Supplementary Figs. 6(a) and 6(b)), This is also the case with -20 dBm, while at the same voltage, the power levels higher than -10 dBm can trigger the phase transition. On the other hand, the power levels higher than -10 dBm are capable of triggering the phase transition even at lower bias voltages, such as 1.690 V (Supplementary Figs. 6(c) and 6(d)). A summary of all these figures combined is found in fig. 2 (d) of the paper.

Supplementary Note 4: CPW S parameters

The spectra of the S parameters for the CPW-based devices are shown in Supplementary Figs. 7(a) and 7(b). By the temperature elevation and therefore the VO_2 interruption becoming more conductive, the spectra become closer to a simple CPW characteristics.

Supplementary Note 5: Noise equivalent power (NEP) evaluation

The Johnson-Nyquist noise in this device has been evaluated according to the insulating phase, where the resistance of the device is maximum 1.4 kΩ. So the upper bound for the thermal noise spectral density would be estimated as 4.8 nV.$Hz^{-1/2}$. But this is a first order estimation of the noise spectral density. To be more inclusive about other

sources of noise such as material drifts, thermal fluctuations, flicker noise, and the noise implied by the setup. In order to address the combined noise in a static measurement, we applied a constant voltage and monitored the current fluctuations over time (200 s). After extracting the product of the current fluctuations and the voltage, we calculate the FFT of the time-domain signal. We normalize the measured values by the resolution bandwidth of the parameter analyzer (250 Hz). Then multiply the normalized power with the frequency difference between two adjacent measurement points to calculate the integral power between the two measurement points: (Therefore, summing up the calculated integral power per frequency step up to the first desired measurement bandwidth, the calculated value represents an integrated output noise power). The NSD (v_N) could be calculated by:

$$v_N = \sqrt{P_{\mathrm{N,out}}.R_\mathrm{L}}$$

Where $P_{\mathrm{N,out}}$ is the output noise power spectrum and R_L is the measurement tool impedance, typically 50 Ω. In order to get the equivalent input noise power, we divide the v_N by the voltage responsivity value (R_V):

$$P_{\mathrm{N,in}} = v_\mathrm{n}/R_\mathrm{V}$$

It is common to use the maximum value of RV. This value is used in the curves of fig. 6 (b) of the paper. Finally, to achieve the NEP we divide this power by the $\sqrt{BW}$. For the case of static detection, a value of 1 Hz is set as the BW.

Supplementary Note 6: Detailed limit of detection (LoD) values

As also indicated in the main text, the minimum detectable signal by the VO_2 two-terminal devices is measured experimentally as 20 nW (fig. 6 (b) in the paper). This is while at any power level the additional uncertaintly of detection should be concidered seperately. We calculated the LoD of the detector based on $3\sigma/R_\mathrm{V}$ at different stages as shown in Supplementary Table 1. The LoD degrades by temperature as the responsivity decays by getting closer to the phase change critical temperature.

Supplementary Note 7: VO_2 film investigation

The X-ray diffraction (XRD) of the VO_2 thin film which is deposited on a Si wafer with a 2 μm SiO_2 buffer layer is shown in Supplementary Fig. 8. The main peak for the (011) phase is well pronounced in this measurement, which is required for the switching behavior. The measurement is repeated with a higher resolution in a smaller range to reduce the noise floor, as shown in the inset of Supplementary Fig. 8.

VO_2 film degradation and aging

The reversible electric-field induced hysteresis loop has shown robustness over millions of non-stop switching cycles in our oscillator measurements. The same devices have been measured over days, showing the same V_IMT and V_MIT threshold voltages.

In case of keeping the devices safe from electrostatic discharge (ESD), they have shown lifetimes of about 2 years since we fabricated them. Also, in case of passivating the VO2 film surface, the degradation is negligible over months. However, most of our devices were not passivated and therefore a degradation in the ON/OFF ratio (between insulating and metallic states) has been observed. On can compare the switching ON/OFF ratio degradation over time according to the resistance characteristics of a VO_2 film depicted in Supplementary Fig.9: Measurement 2 is taken about 2 months after Measurement 1 on the same VO_2 film, and Measurement 3 is taken after almost a year.

This is while the threshold voltages are slightly affected and therefore our method of sensing barely suffers from that: In the static approach (figs. 2(e) and 4(a)), the resistivity in either of the states is not even used in the detection method. In the oscillator approach, the resistivity could influence the time constants of the circuit, but again, we refer to the relative shift in the frequency of oscillations with respect to a baseline which is the free running with no external power exposed. So the sensor remains functional and only re-calibrations are needed.

Supplementary Note 8: Fabrication Process

A layer of 2 μm thermal oxide was grown on the surface of a high resistivity float zone (HRFZ) Silicon wafer. Then using pulsed laser deposition (PLD), we synthesized a 100-nm-thick VO_2 film on top of SiO_2. The VO_2 film was patterned using photolithography on a 1.4 μm layer of negative tone AZ nLof 2020 resist, developed in AZ 726 MIF developer. Wet etching of VO_2 was done in diluted 1:6 Cr etch solution. We stripped the resist by Oxygen plasma

and putting it in remover 1165 for 30 minutes. For the second layer, 15 nm Cr was sputtered on the patterned substrate as an interface layer and the process was followed by the sputtering of 150 nm Au. The photolithography of the second layer was done with the same resist coating parameters and stripping method. The connections and the 2-terminal devices were completed by dry etching of the sputtered metal using a broad beam of Argon ions in an ion-beam-etcher. Resist stripping was done using the same method as the first lithography. The process flow is illustrated in Supplementary Fig. 10.

Supplementary References

1. Jerry, M., Shukla, N., Paik, H., Schlom, D. G. & Datta, S. Dynamics of electrically driven sub-nanosecond switching in vanadium dioxide. In *2016 IEEE Silicon Nanoelectronics Workshop (SNW)*, 26–27, DOI: 10.1109/SNW.2016.7577968 (2016).

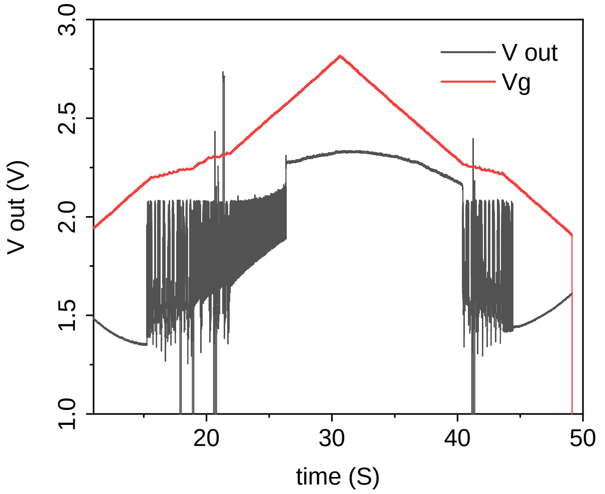

Supplementary Figure 1. Locating the operational region of the spike generator. The gate voltage of the transistor is swept to find the proper bias regions for oscillation conditions.

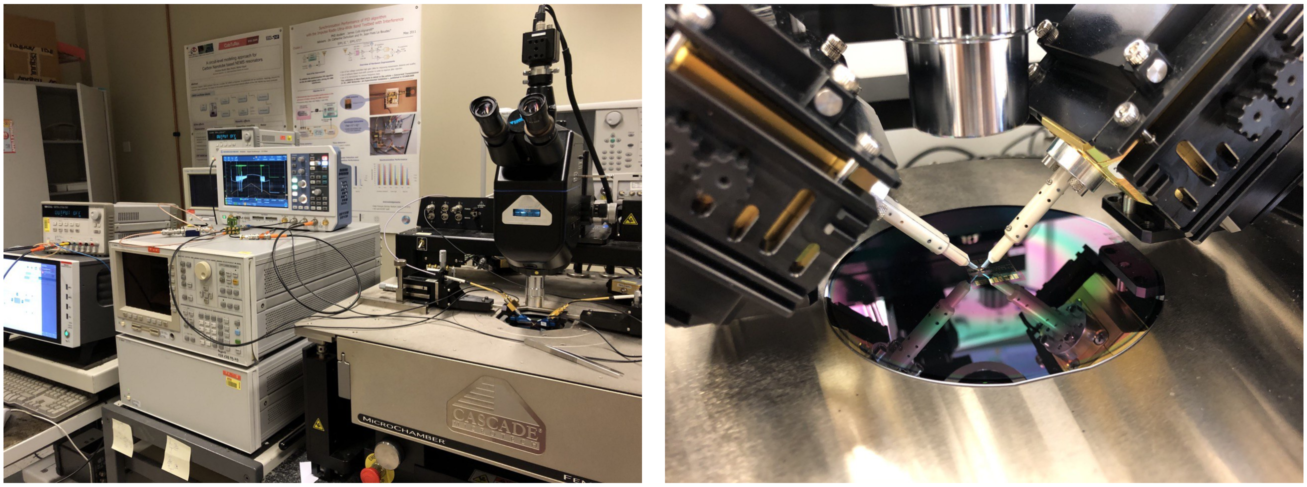

(a) (b)

Supplementary Figure 2. Characterization setup for the spike generator. (a) Experimental setup for astable circuit, (b) On-wafer VO_2 devices along with the millimeter-wave (mm-wave) GSG infinity probes

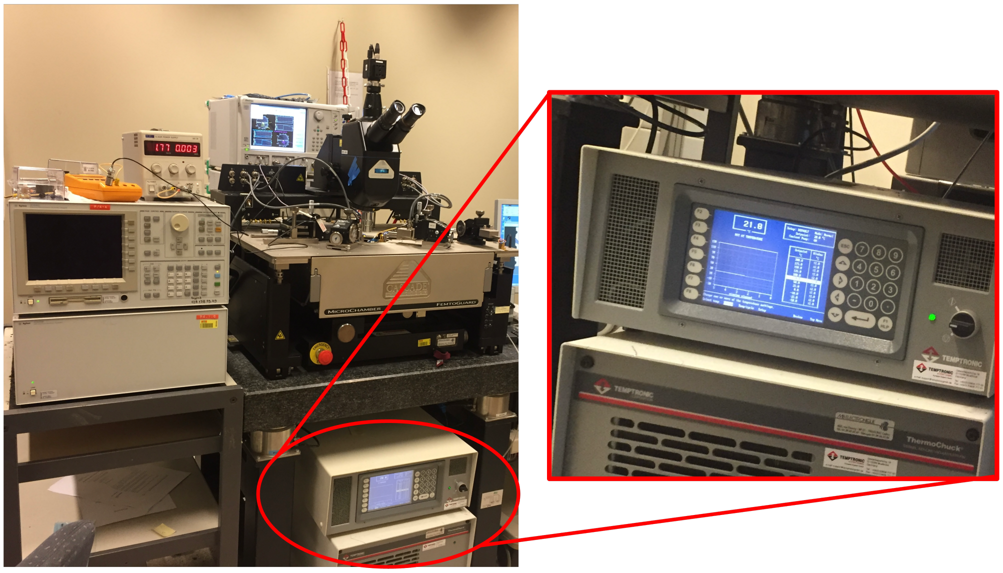

Supplementary Figure 3. **Temperature reservoir in the setup**

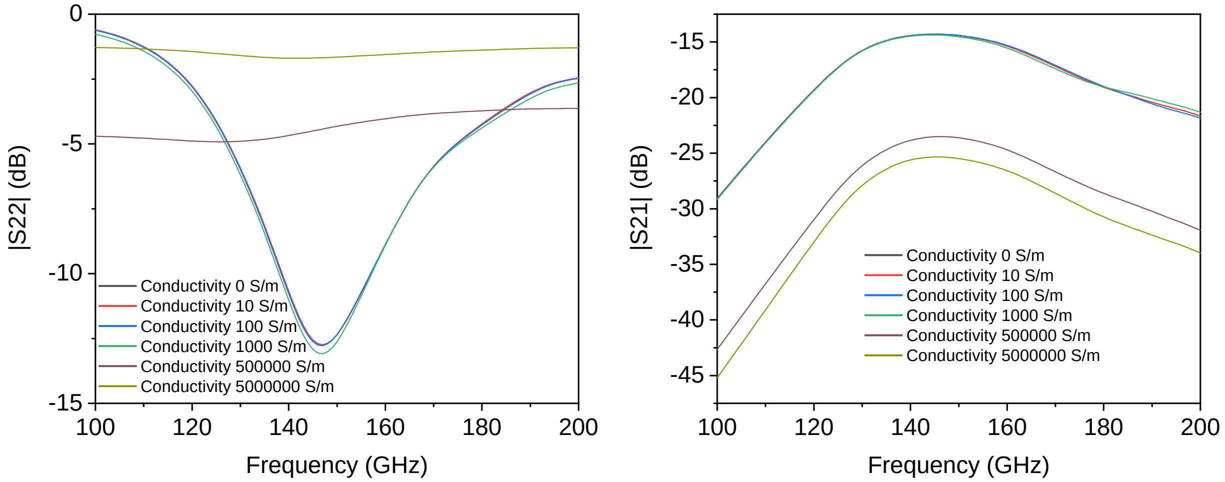

(a) (b)

Supplementary Figure 4. **Finite element method (FEM) simulations for the S parameters of the coupled antennas with respect to VO_2 conductivity**. (a) reflection, (b) transmission.

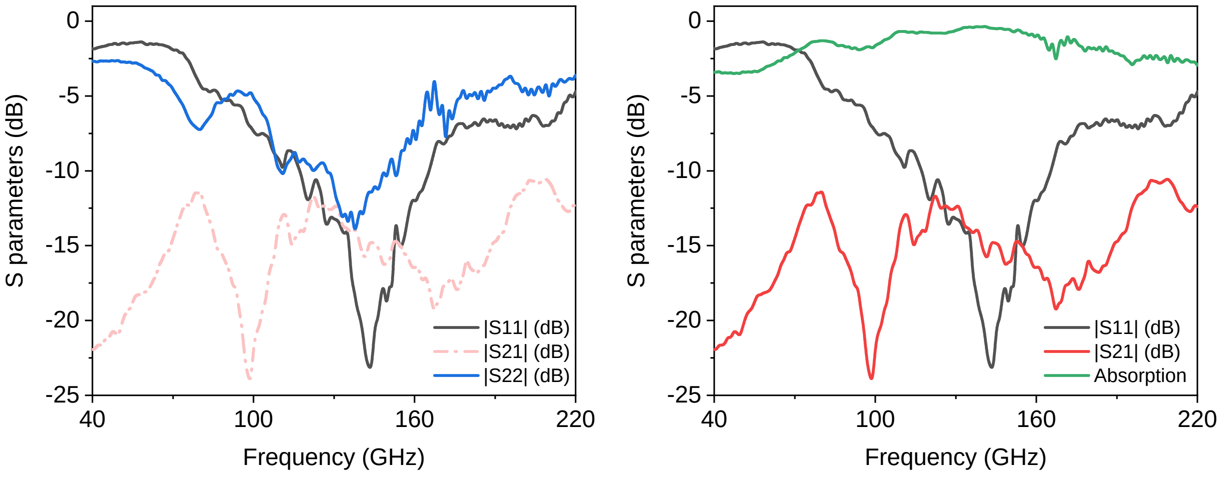

(a) (b)

Supplementary Figure 5. Measured S parameters for the coupled antennas (a) all the S parameters together. The peaks of $|S_{11}|$ and $|S_{22}|$ are not at the same frequencies. (b) the absorption of the emitted signal by the ambiance calculated by $1 - |S_{11}|^2 - |S_{11}|^2$

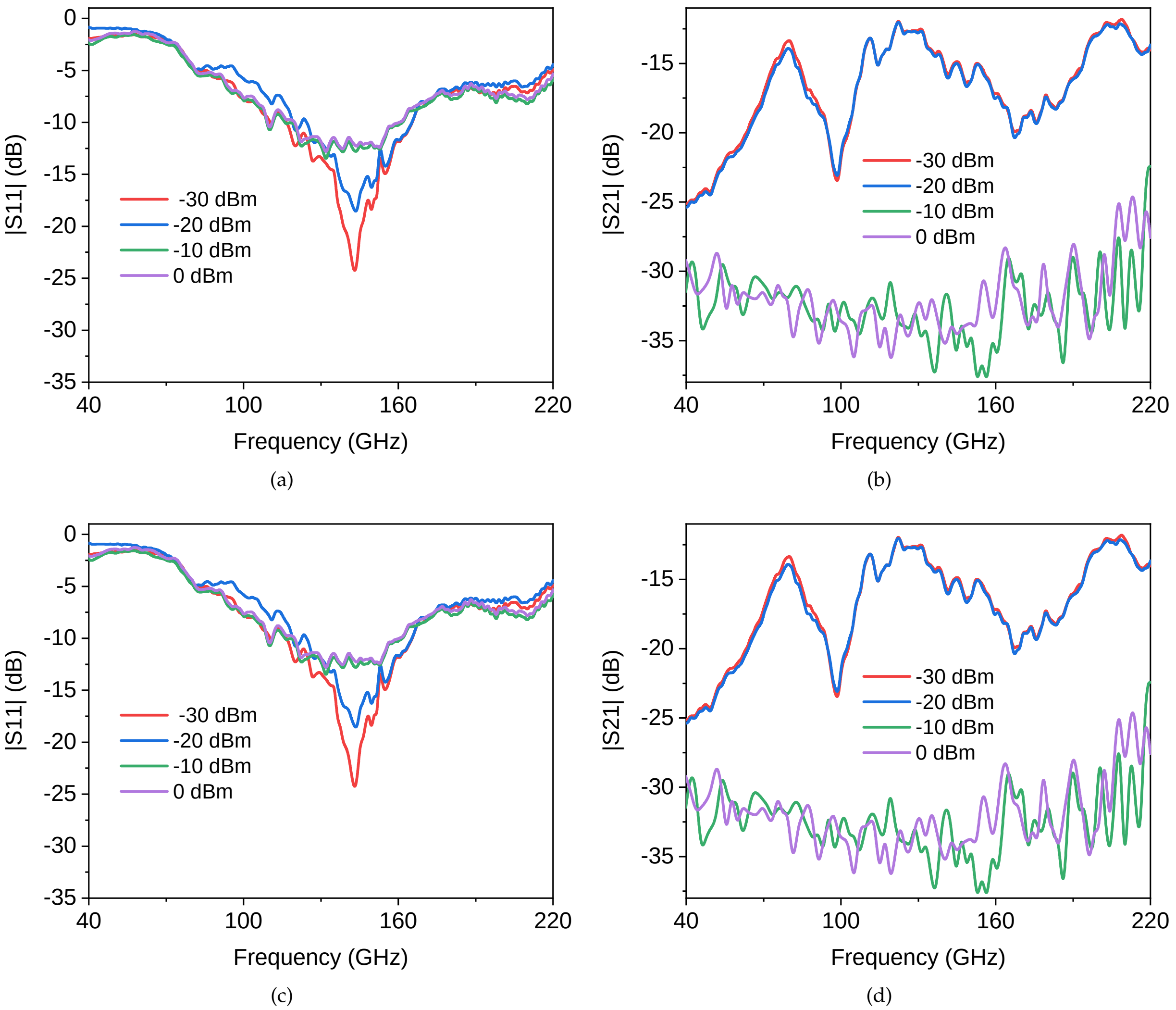

(a) (b) (c) (d)

Supplementary Figure 6. Measured S parameters for the biased antennas at different voltage levels (a) $|S_{11}|$ for the bias voltage of 1.705 V. (b) $|S_{21}|$ for the bias voltage of 1.705 V. (c) $|S_{11}|$ for the bias voltage of 1.690 V. (d) $|S_{21}|$ for the bias voltage of 1.690 V.

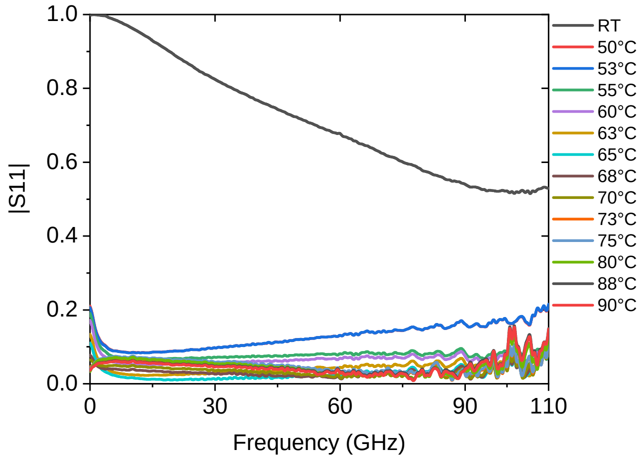

(a)

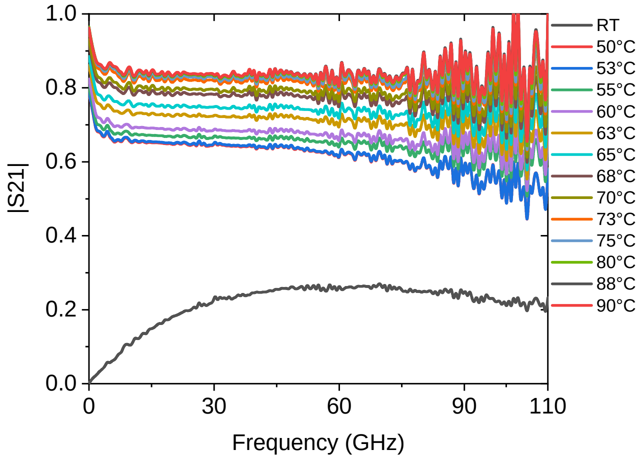

(b)

Supplementary Figure 7. S parameters spectra by temperature for the interrupted CPW. (a) $|S_{11}|$, (b) $|S_{21}|$

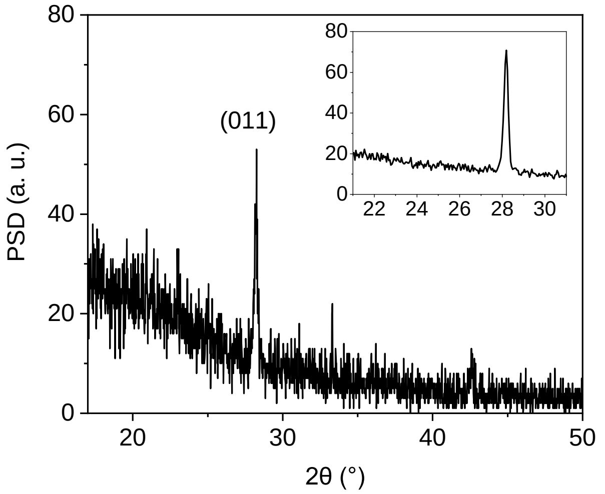

Supplementary Figure 8. VO_2 film investigation by XRD. measured for a 100-nm-layer of VO_2 thin film on top of Si/SiO_2 substrate, inset: the main peak with higher resolution

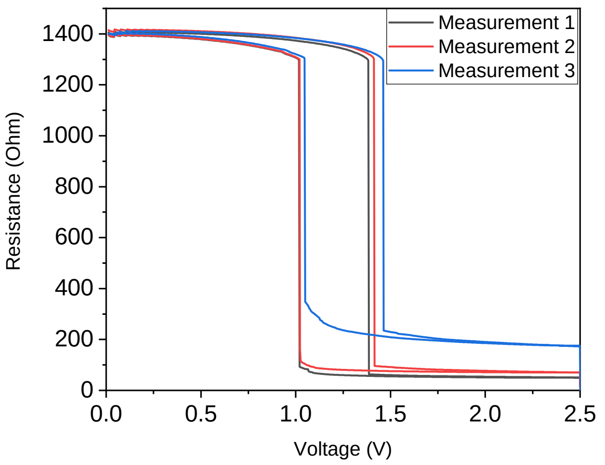

Supplementary Figure 9. Sample measurements of MIT switching degradation over time. Measurement 2 is taken about 2 months after measurement 1 on the same VO_2 film, and measurement 3 is taken after almost a year.

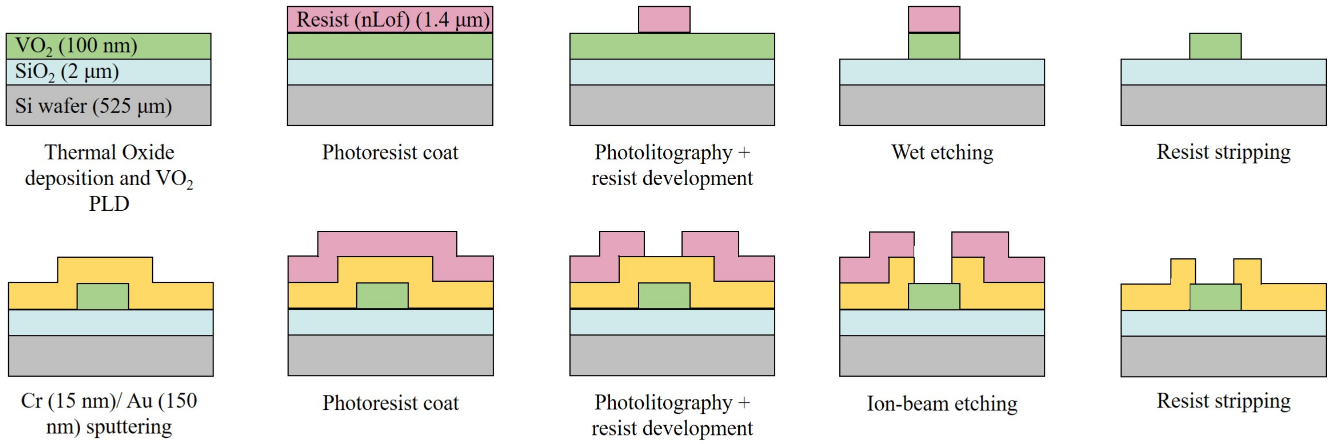

Supplementary Figure 10. Summarized process flow of the VO_2 two-terminal devices. Including the antennas or CPWs patterning

Configuration	-30 dBm	-20 dBm	-10 dBm	0 dBm
CPW (RT)	67.8	245.7	758.5	2239.2
CPW (40°C)	124.9	562.4	1703.2	5980.1
CPW (50°C)	2621.6	10068.7	43736.8	185312.
Antennas (RT)	959.2	3736.3	11332.1	33832.3

Supplementary Table 1. The LoD of the detector in different temperatures, based on $3\sigma/R_{\mathrm{V}}$ of the IMT threshold voltages statistics. All the units in nW.
